# Stable fly activity is associated with dairy management practices and seasonal weather conditions

**DOI:** 10.1371/journal.pone.0253946

**Published:** 2021-07-28

**Authors:** Wagdy R. ElAshmawy, Essam M. Abdelfattah, Deniece R. Williams, Alec C. Gerry, Heidi A. Rossow, Terry W. Lehenbauer, Sharif S. Aly

**Affiliations:** 1 Veterinary Medicine Teaching and Research Center, School of Veterinary Medicine, University of California Davis, Tulare, California, United States of America; 2 Department of Internal Medicine and Infectious Diseases, Faculty of Veterinary Medicine, Cairo University, Giza, Egypt; 3 Department of Animal Hygiene and Veterinary Management, Faculty of Veterinary Medicine, Benha University, Qalyubia, Egypt; 4 Department of Entomology, University of California, Riverside, California, United States of America; 5 Department of Population Health and Reproduction, School of Veterinary Medicine, University of California, Davis, California, United States of America; Beni Suef University Faculty of Veterinary Medicine, EGYPT

## Abstract

Stable flies (*Stomoxys calcitrans)* are blood-sucking insects commonly associated with cattle production systems worldwide and are known to cause severe irritation to cattle due to painful bites. Cattle react to biting stable flies with an aggregating behavior known as bunching. Bunching behavior reduces grazing or feed consumption and thus reduces cattle productivity and welfare. Cattle’s fly-repelling behaviors include foot stomping, head tossing, tail switching and skin twitching. A longitudinal study was conducted in 2017 on 20 California dairies (average lactating herd size = 2,466 (SE±28.392)) during the stable fly season from April to July. The study objectives were to estimate the association between environmental factors and dairy characteristics including facility design, feed and manure management, total mixed ration (TMR) components fed to cattle, and operational pest management procedures and the outcome stable fly activity on California dairies. Stable fly activity was measured by counting stable flies on cow forelimbs (leg count) and on Alsynite traps (trap count) over the 13-week study period. Weekly leg counts were performed for cattle in lactating cow pens (31 pens from 10 study dairies) with counts made during the morning (AM) and again during the afternoon (PM). Trap counts were performed on all 20 study dairies. Data were analyzed using linear mixed models which revealed temporal variation in the average leg and trap counts with stable fly activity increasing from May to June and then decreasing to the lowest activity in July. Leg counts were higher during the afternoon compared to morning. Ambient temperatures ≤30⁰C and relative humidity (RH) measurements <50% were associated with higher leg and trap counts. Traps located at the periphery of study dairies had higher stable fly counts compared to traps located in the interior of the dairy. Cow pens with trees on the periphery had higher leg counts in comparison to pens away from trees. Specific TMR components were associated with both leg and trap counts. Dairies feeding by-products including almond hulls, wet distillers’ grain, fruits, and vegetables had higher trap counts compared to dairies that did not feed these ingredients. At the pen level, pens with rations that contained straw had lower average leg counts compared to pens fed with rations that did not contain straw. A similar association was observed for pens with rations that contained wheat silage when ambient temperatures were ≤30⁰C. In contrast, pens with water added to the TMR while the RH was ≥50% had higher average leg counts compared to pens without water added to the TMR. Dairies that applied insecticides for fly control to their entire facility had lower trap counts compared to dairies that did not apply insecticides. Stable fly activity measured on California dairies using leg and trap counts varied according to the month, environmental factors, pen surroundings, trap location, TMR components, and insecticide use.

## Introduction

The stable fly (*Stomoxys calcitrans*) is a cosmopolitan blood-feeding fly that is distributed globally wherever humans keep domesticated animals [1]. Due to their very painful bites, stable flies influence the behavior, health, productivity, and welfare of cattle [2–8]. Fly-defensive behaviors of cattle bitten by stable flies include tail switching, foot stomping, head tossing, skin twitching, and ear flicking [9–12]. In addition, cattle may group in response to stable fly attacks in an aggregating protective behavior, commonly known as “bunching” [9, 13–15]. Bunching can contribute to reduced feed intake, weight gain, and milk production by individual cattle [15]. Cattle bunching is positively associated with the number of stable flies biting cattle on the front legs [16]. The estimated average loss to U.S. cattle industries induced by *Stomoxys calcitrans* was $2.2 billion per year in 2009, with $360 million lost from the dairy industry alone [4].

Manure handling, feeding practices and ration management within the commodity barn (the location on a dairy where ration components are stored and mixed prior to feeding cattle) may play an important role in the abundance of stable flies on the dairy [17]. Stable flies develop in areas with decaying organic matter such as aged cattle feces, decomposed plant material including straw, hay, alfalfa, silage, and unmarketable vegetables and fruits [1, 17–20]. The decaying organic matter can be found on the dairy around the commodity barns, cow pens and manure collection sites [17–19]. Furthermore, the stable fly’s preferences amongst these sites for oviposition may rely on environmental factors such as moisture, temperature, organic matter type and pH [21].

In California, adult stable fly populations begin to increase by mid-late April with a peak in adult activity in late May through early June [21, 22]. Stable fly abundance is associated with variation in rainfall, ambient temperature and relative humidity (RH) during the fly season [9, 22–29]. Temperature and precipitation alone are reported to account for 72% of the variation of stable fly counts on traps in Nebraska [25] and there is a significant positive association between March rainfall and later stable fly abundance, at least in Southern and Central California [23]. Surveys on California dairies show that stable flies predominantly deposit their eggs in aged manure associated with cattle pens, particularly in areas where manure builds up beneath fence lines [17]. While previous studies identified development sites for stable flies suggesting management practices that may be related to stable fly intensity[17, 20, 30–32], specific characteristics of dairy facility design, total mixed ration (TMR) components, and manure management were not explored. Hence, studies on the association between TMR components (animal feed), facility design, feed and manure management and cow breed on stable fly abundance on dairies are needed to identify opportunities for control of stable flies on modern dairies.

The objectives of our study were to: 1) estimate stable fly activity on California dairies during the peak stable fly season using sticky Alsynite traps and visual counts on cow forelimbs; and 2) determine the association between environmental factors (ambient temperature, RH), TMR components, facility design, feed, and manure management, fly control practices, and cow breed on stable fly activity on California dairies.

## Materials and methods

University of California Davis Institutional Animal Care and Use Committee approved the current study (protocol number 19088). The study’s survey tool is exempted by the Institutional Review Board (protocol number 1476320–1).

### Study design and herds

Data for this research was part of a larger study that investigated the epidemiology of cattle bunching [16]. Briefly, the current study data were collected from 20 dairies in Tulare and Kings Counties between April 26^th^, 2017 and July 31^st^, 2017. Herd selection was based on owner willingness to participate, location, herd size, pen designs, cow breeds and the use of fly control.

#### Herd demographics

Cow breeds on the study dairies were Holstein (16 herds), Jersey (2 herds), or mixed Jersey and Holstein (2 herds) with an overall mean herd size of 2,466 (SE±28.392) and an annual rolling herd average (365-day average milk produced per cow) of 11,948 kg (SE± 51.559). Lactating cows were milked on the 12 study dairies twice per day, on 7 dairies three times per day, and on one dairy either twice or three times per day depending on cow parity. The lactating cows on 8 dairies were housed in freestall pens with or without exercise area, on five dairies in open lot pens, and on 7 dairies in mixed design with both freestall and open lot pens.

An on-farm enrollment survey was conducted in person with the herd owner or manager prior to study commencement. The complete survey is available online [16]. Briefly, the survey’s 151 questions were divided into six sections: 1) herd information; 2) facility design; 3) management of animal feed, bedding and manure; 4) cow cooling; 5) presence or absence of calves on the dairy; and 6) fly control methods. Following the enrollment survey, study personnel conducted a walkthrough of the farm to observe and record information about pen design (free stall pen with or without exercise area, dry lot or bedded pack for each lactating, dry and late gestation pen), barn characteristics (roof material, height and width), environment surrounding both the dairy and the pens (adjacent crops, tree crops or tree lines, or main road), commodity barn characteristics (type of silage storage, feeding vegetables or fruits, and adding water, whey or molasses to the TMR), cow cooling system (use of fans or soakers and whether leakage was observed from the soakers or at the water troughs), and waste management (manure accumulation around pen fences, feed curbs, leftover TMR swept off the feed manger–also known as feed refusal, and wet spots). Finally, data on the components of the TMR fed to the adult cows in each pen on the study dairies during the study period were retrieved from the herds’ ration software programs (Feed Watch, Valley Agriculture Software, Tulare, CA; and EzFeed, Amelicor, Utah).

#### Stable fly count on cows (leg counts)

Due to feasibility, leg counts were recorded on only 10 of the 20 study dairies selected based on inclusion of different cow breeds, herd sizes, pen designs, and fly control methods. Leg counts were recorded as previously described [9, 16]. Briefly, trained study personnel recorded the number of stable flies observed on the forelimbs of 15 cows not aggregating in a bunch in three lactating pens representing different locations on the 10 dairies. One of the 10 dairies recorded daily milk weights and leg counts were recorded in four pens on this dairy to estimate the impact of bunching on milk production; results to be reported in a separate publication. Stable fly counts were performed with the observer outside the pen using 10 x 42 binoculars (Nikon Prostaff® 3S, Tokyo, Japan) to view the front legs of an individual cow. Stable flies below the elbow and oriented in a heads-up position (biting position) on the outside of one forelimb and the inside of the other were counted, the sum of counts from a side from each leg estimate the stable fly count per leg, here onwards referred to as leg count [9, 10, 33]. At each dairy, stable flies were counted once weekly between 9:00 to 11:00 AM (morning, AM) and again between 12:00 and 2:00 PM (afternoon, PM) by two trained study personnel. Ambient temperature and RH were recorded before every pen count using a smart phone application (AccuWeather®, American Media Company, State College, Pennsylvania). The mobile application reports temperature and humidity recordings from the nearest weather station which in the case of our study dairies recordings were from between two weather stations (WBAN: 23149 and WBAN:93144) and all the study dairies were within 20 miles from these stations.

#### Stable fly trap counts

Stable fly activity was recorded on the 20 study dairies using Alsynite traps as described in El-Ashmawy et al., (2019). Briefly, a total of 5 Alsynite traps (Biting Fly Trap®, Olson Products Inc, Medina, OH) were placed on each dairy at locations to optimize the exposure of these traps to different landmarks on or adjacent to the dairy such as trees, roads, decomposing vegetation, silage or manure mounds. For each study dairy, four traps were placed at the periphery of the dairy and one trap was placed at the interior of the dairy to capture the variability in stable fly count among different locations. The number of stable flies on each trap was recorded weekly for 13 weeks from May 1^st^ through July 31^st^, 2017. Each week, the sticky sheets on the traps (Sticky Sleeve^TM^, Olson Products Inc, Medina, OH) were replaced and the number of stable flies on the trap was recorded.

### Statistical analyses

The computer software used for all statistical analyses was Stata 15.1®.

#### Modeling stable fly activity

Both leg and trap counts were modeled guided by a causal diagram of explanatory risk factors recorded during the enrollment survey and the dairy walkthrough [16]. As our interest was to estimate the variation of stable fly activity at the pen level, the mean leg count per pen (mean count of 15 cows’ forelimbs) was used in our models. Mean leg count and individual trap counts were transformed to log_10_ after assessing the Q-Q plot for normality. General linear mixed models (LMM) were used to model variation in the log_10_ mean leg count and the log_10_ stable fly trap counts.

#### 1. Modeling log_10_ of the mean leg count

Eq 1 summarizes the model used to estimate the regression coefficients for the association between the explanatory variables (X) and the outcome (*y*_*mijkl*_) log_10_ of the mean leg count observed twice per day for 15 cows per pen in 31 lactating cow pens from 10 study dairies.


$$y_{mijkl}=\beta_{0}+\beta X+t_{m}+u_{mi}+v_{mij}+w_{k}+z_{kl}+e_{mijkl}$$ Eq [1]

*Random effects*. The random effects were specified as described in [16]. Specifically, for each outcome there were 5 random effect variables included in the model: observer, dairy, pen, week, and observation time (AM or PM). Dairies were nested under observer and pens were nested within the dairy, while observation time was nested within week. Pens were also crossed with week and observation time. The dairies were nested within observers*; m* = 2 {observer 1, observer 2}. On dairy *i*,*i* = {1,2,3,4,5,6,7,8,9,10}, were pens *j*, where for *i* = 1 *to* 9,*j* = {1,2,3} and for dairy *i* = 10,*j* = {1,2,3,4}. During week *k*, *k* = {1,2,..,13}, mean leg count was recorded at time *l*, where *l* = {*AM*, *PM*}. The random effects for observer, dairy, pen, week, and daytime were *t*_*m*_, *u*_*i*,_ *v*_*j*_, *w*_*k*_ *and z*_*l*_, respectively. All random effects (*t*_*m*_, *u*_*i*,_ *v*_*j*_, *w*_*k*,_ *z*_*l*_,) and the residual error (*e*_*mijkl*_) were assumed to be normally distributed with a mean = 0 and variances σ^2^_t_, σ^2^_u,_ σ^2^_v,_ σ^2^_w,_ σ^2^_z_, and σ^2^, respectively.

*Fixed effect variables*. The model included an intercept (*β*_0_) and the remaining fixed effect variables (*βX*), the latter specified as either dairy or pen level variables. Variables obtained from the survey and walkthrough were each explored using univariate models that included the random effect variables. Ambient temperature and relative humidity used in the model were the values recorded before each pen count. A Box-Tidwell test showed that the association between each of the variables ambient temperature and RH and the outcome mean leg count were non-linear [34]. Therefore, temperature and RH were explored in the models as categorical variables. Optimum categories of the ambient temperature and relative humidity variables were determined using box plots and exploratory analyses specifically the average, median and quartiles. The best categorization for ambient temperature was ≤ 30 ⁰C and > 30 ⁰C, and for RH was < 50% and ≥ 50% which were also confirmed using the Akaike Information Criterion (AIC) [35].

#### 2. Modeling the log_10_ of the stable fly trap counts

A LMM was specified to model log_10_ of stable fly trap count (*y*_*ijkm*_) on the 20 study dairies each with 5 fly traps, as in Eq (2).


$$y_{ijkm}=\beta_{0}+\beta X+u_{i}+v_{ij}+w_{k}+t_{m}+e_{ijkm}$$ Eq [2]

*Random effects*. The random effects were specified as described in [16]. Specifically, for each outcome there were 4 random effect variables in the model including, dairy, trap number, week, and observer. Traps were nested within the dairy and were crossed with week and observer. On dairy *i*,*i* = {1,2,…,20}, were traps *j*, where *j* = {1,…,5}, for the entire study period, week *k*,*k* = {1,2,…,13}, and observer *m* where*; m* = {1,…,6}. Where the random effects for dairy, pen, week, and observer were *u*_*i*_, *v*_*j*_, *w*_*k*_ *and t*_*m*_, respectively. All random effects (*t*_*m*_, *u*_*i*_, *v*_*j*_, *w*_*k*_) and residual error (*e*_*ijkm*_) were assumed to be normally distributed with mean = 0 and variances σ^2^_t_, σ^2^_u,_ σ^2^_v,_ σ^2^_w,_ and σ^2^, respectively.

*Fixed effect variables*. The fixed effect variables were dairy level factors, environmental variables, and trap related factors. All the variables from the dairy survey and walkthrough were entered first in univariate models with the random effect variables. The trap count represents the number of flies per trap for the whole week, hence, it was modeled using the weekly average temperature and relative humidity. The weekly average temperature and humidity estimate was obtained by averaging the daily estimates for the previous 7 days. The daily estimates were the measurements recorded during each stable fly count on cow legs, twice a day between 9:00–11:00 and again between 12:00–2:00 PM, every day starting May 1^st^ until July 30^th^. Given that temperature and humidity were measured twice per day, separate models were explored to model the weekly trap count as explained by temperature and humidity estimates recorded in the AM and PM. Finally, a third model was explored to predict the weekly trap counts as explained by the weekly temperature and humidity estimates averaged over AM and PM.

Significant variables in the univariate models were fitted in the final model, and variables that were no longer significant were excluded from the final model.

#### Selection of the final models

Models were fitted for each variable separately while including the respective random effect structure described above. The final models were selected using a manual backward process [36, 37]. During the variable selection and model building, confounding was assessed using the method of change in estimates [38] and two-way interactions were tested using significance testing. The AIC estimate was used to compare competing models with lower values denoting better model goodness of fit [35]. A 5% level of significance was used in all models.

## Results

### Descriptive statistics

#### Temperature and relative humidity

Temperature recordings observed between 9:00–11:00 AM and 12:00–2:00 PM during the study period (May 1st to July 30th, 2017) had a mean of 23.30 ⁰C (SE ±0.249) with a minimum of 11.11⁰C and maximum of 34.44 ⁰C for AM recordings; and a mean of 28.91 ⁰C (SE±0.281) with a minimum of 15.55 ⁰C and maximum of 41.11 for PM recordings. The temperature recordings averaged over both AM and PM periods was 26.11⁰C (SE ±0.212).

For relative humidity recordings observed between 9:00–11:00 AM and 12:00–2:00 PM during the study period (May 1st to July 30th, 2017) had a mean of 53.11% (SE±0.005) with a minimum of 22% and maximum of 86% for AM recordings; and 38.66% (SE±0.004 with a minimum of 19% and maximum of 76% for PM recordings. The relative humidity recordings averaged over both AM and PM periods was 45.9% (SE±0.004).

#### Leg count

**Fig 1:**
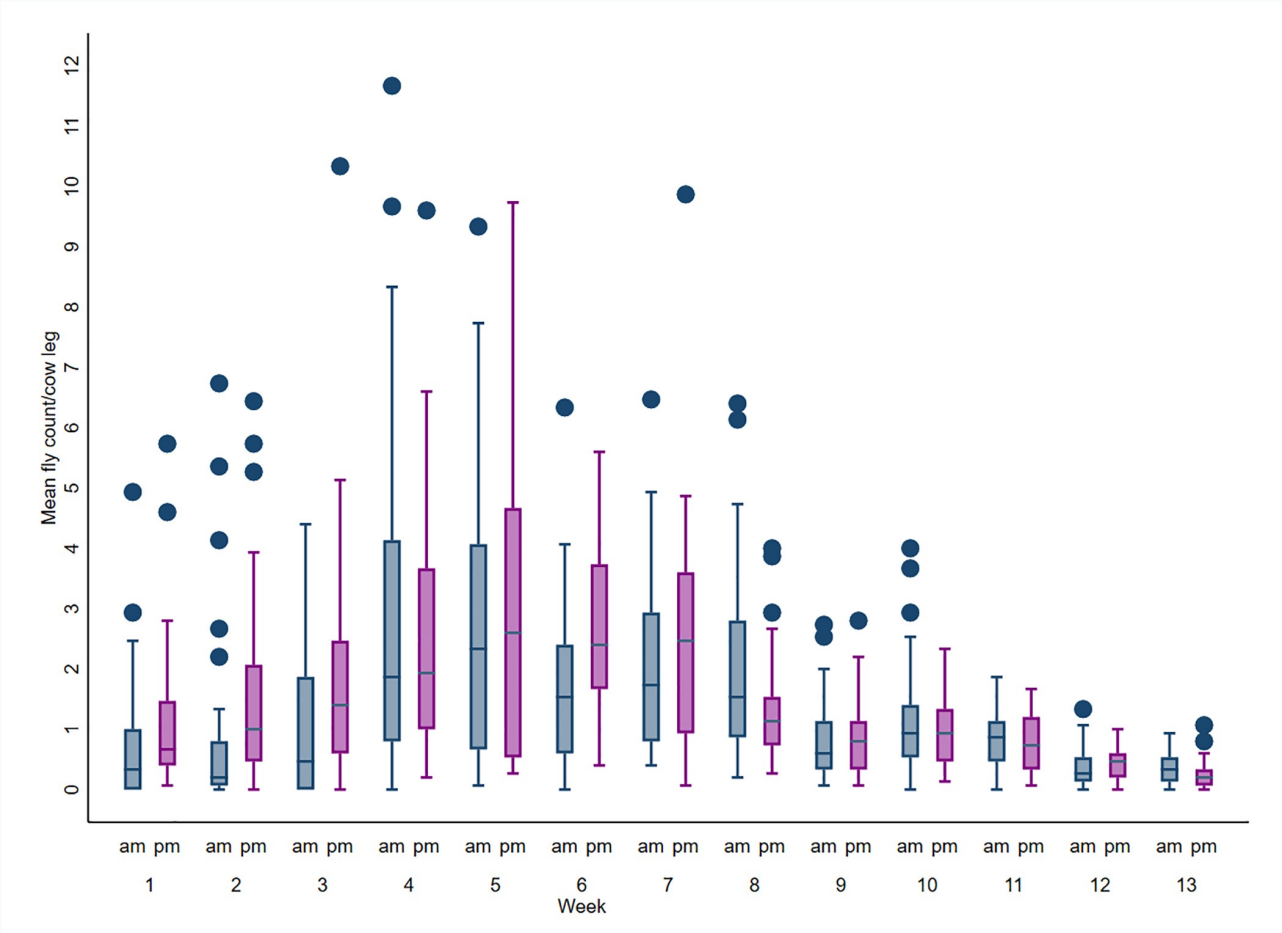
Boxplot of the mean weekly leg counts observed during morning (AM) and afternoon (PM) on the 10 study dairies over 13 weeks from May 1st to July 31st, 2017. The upper and lower horizontal lines of the box represent the 25^th^ and 75^th^ percentiles respectively, the horizontal midline represents the median, the upper and lower horizontal whiskers lines are the upper and lower limits, and the dots are the outliers.

The mean weekly leg count for all 31 lactating cow pens on the 10 study dairies from May 1^st^ to July 31^st^, 2017 are depicted in Fig 1. The greatest counts were recorded from weeks 4–8 which represent the peak of the stable fly season (late May through late June) during the study period. Mean leg count increased from May (1.58± SE 0.11 flies/cow forelimb) to peak in June (2.06± SE 0.11 flies/cow forelimb) before decreasing to the lowest counts in July (0.65 ± SE 0.04 flies/cow forelimb) on the study dairies (Fig 1). Specifically, the mean leg count peaked in the first week of June while the minimum count was recorded in the last week of July.

Results of the model predicting log_10_ of mean leg count as explained by the management and environmental factors are presented in Table 1. Variances (SE) in the mean leg count attributable to observer, dairy, pen, week, and observation time were 0.004 (0.005), 0.001 (0.001), 0.006 (0.003), 0.028 (0.004), and 0.001 (0.001), respectively. There was a significant difference (*P* < 0.01) in the mean leg count by month (May, June and July), with leg count being greatest in June (1.62 flies/cow forelimb) followed by May (1.17 flies/cow forelimb) and then July (0.87 flies/cow forelimb). The mean leg count was greater (*P* < 0.01) in PM (1.02 flies/cow forelimb) relative to AM (0.87 flies/cow forelimb). Leg counts were also greater (*P* < 0.01) when ambient temperature was ≤ 30°C (1.38 flies/cow forelimb) in comparison to >30°C (0.87 flies/cow forelimb), and greater (*P* < 0.01) when RH <50% (0.87 flies/cow forelimb) in comparison to RH ≥ 50% (0.64 flies/cow forelimb). Leg counts recorded in lactating cow pens with adjacent trees (1.07 flies/cow forelimb) were significantly greater (*P* = 0.03) than counts in pens lacking adjacent trees (0.87 flies/cow forelimb).

**Table 1:** Linear mixed model to estimate the association between the outcome log_10_ mean leg count and explanatory variables study month, observation time, environmental factors, and addition of water, wheat silage, or straw to total mixed ration (TMR).

Variable	Levels	Coefficient	Standard error	P-value	95% CI	
					Lower	Upper
Intercept		-0.06	0.063	0.37	-0.18	0.07
Month	July	Referent				
	May	0.13	0.029	<0.01	0.07	0.19
	June	0.27	0.028	<0.01	0.21	0.32
Observation time	AM	Referent				
	PM	0.07	0.021	<0.01	0.03	0.12
Relative humidity (%)	<50%	Referent				
	≥50%	-0.13	0.028	<0.01	-0.18	-0.07
Addition of water to TMR	No	Referent				
	Yes	0.03	0.042	0.50	-0.05	0.11
Interaction Term: Addition of water to TMR X relative humidity ≥50%		0.10	0.035	0.01	0.03	0.17
Ambient temperature (°C)	>30°C	Referent				
	≤30°C	0.20	0.029	<0.01	0.14	0.26
Addition of wheat silage to TMR	No	Referent				
	Yes	-0.01	0.056	0.80	-0.12	0.095
Interaction Term: Addition of wheat silage to TMR X ambient temperature ≤30°C		-0.13	0.037	<0.01	-0.20	-0.06
Addition of straw to TMR	No	Referent				
	Yes	-0.09	0.046	0.04	-0.19	-0.003
Presence of trees adjacent to the pen	No	Referent				
	Yes	0.09	0.041	0.03	0.01	0.17

Addition of straw to the TMR of lactating pens was significantly associated (*P* = 0.04) with lower mean leg counts (0.70 flies/cow forelimb) in comparison to pens without straw added to their TMR (0.87 flies/cow forelimb). In contrast, addition of wheat silage to the TMR was only significantly (*P* <0.01) associated with lower leg counts when ambient temperatures were ≤ 30°C (1.00 flies/cow forelimb) compared to no wheat silage in TMR at the same temperatures (1.38 flies/cow forelimb). Addition of water to the TMR was only significantly (*P* <0.01) associated with a higher mean leg count when RH was ≥ 50% (0.87 flies/cow forelimb) compared to pens without water added to their TMR at the same RH (0.64 flies/cow forelimb). The remaining explanatory variables were not significantly associated with leg count and hence dropped from the model.

#### Stable fly trap counts

**Fig 2:**
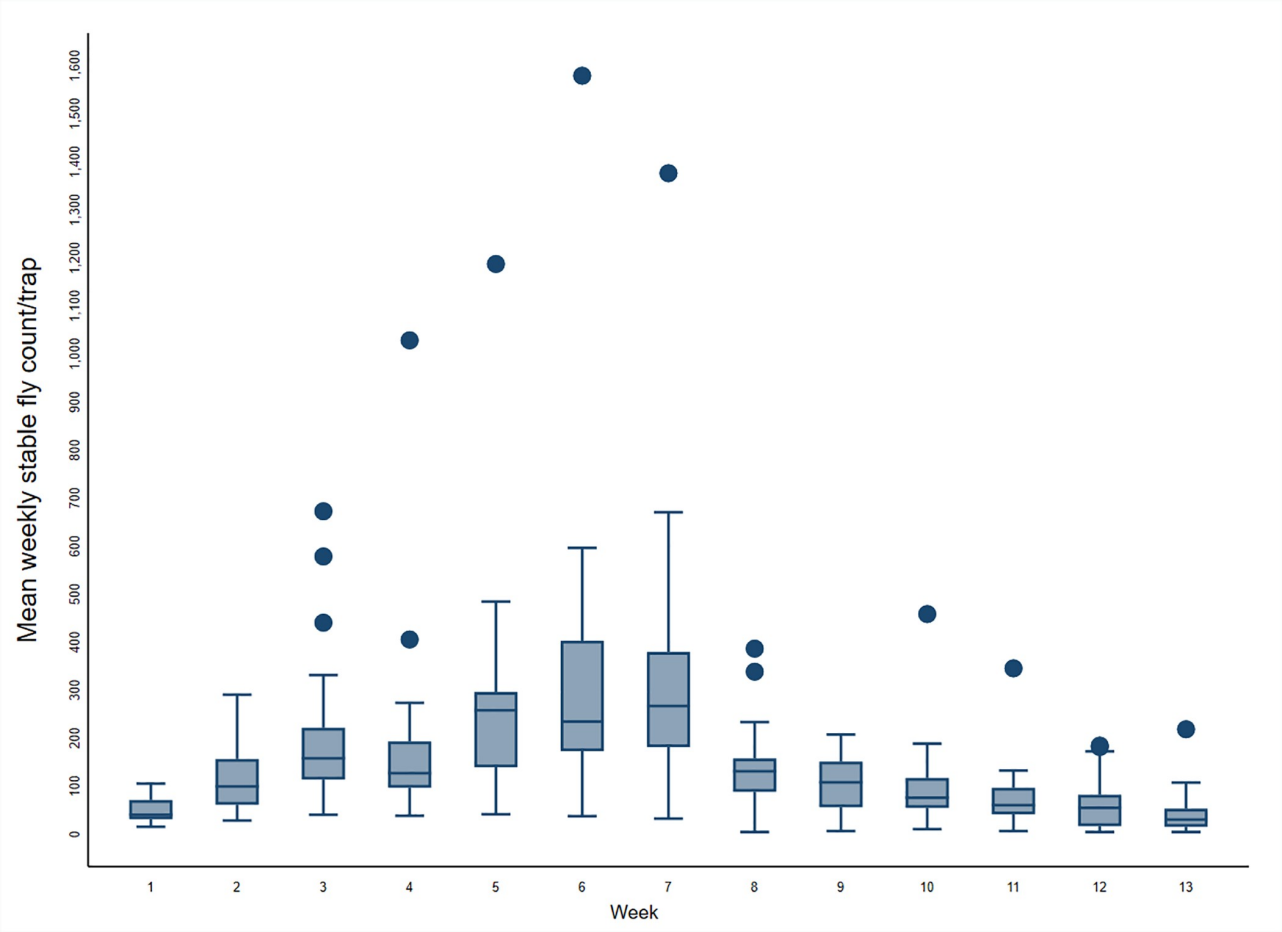
Boxplot of the mean weekly stable fly trap counts using five Alsynite traps on each of 20 study dairies over 13 weeks from May1st to July 31st, 2017. The upper and lower horizontal lines of the box represent the 25^th^ and 75^th^ percentiles respectively, the horizontal midline represents the median, the upper and lower horizontal whiskers lines are the upper and lower limits, and the dots are the outliers.

**Fig 3:**
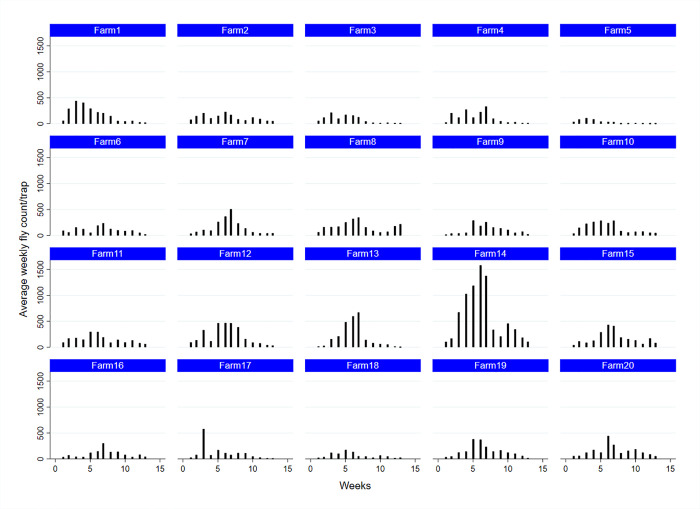
Weekly mean stable fly trap count using five Alsynite traps on each of 20 study dairies over 13 weeks from May1st to July 31st, 2017 during the peak stable fly activity season in the San Joaquin Valley of California.

Weekly stable fly trap counts from each study dairy over 13 weeks from May 1^st^ to July 31^st^, 2017 increased from May (142.01± SE 8.95 flies/trap/week) to peak in June (272.15± SE 16.98 flies/trap/week) before decreasing in July (77.74 ± SE 4.65 flies/trap/week) (Fig 2). This activity pattern was similar for most of the study dairies (Fig 3). Specifically, the mean trap counts peaked in the second week of June and the minimum count was recorded in the last week of July.

The weekly average of both AM and PM temperature and relative humidity had better model fit for predicting the weekly log_10_ of the stable fly trap counts compared to separate AM or PM estimates of both environmental measures (Table 2). Due to the small variance value for the random effect of dairy and hence inestimable standard error, dairy was not included as a random effect. Variances (SE) in the mean trap count due to trap, week and observer were 0.04 (0.011), 0.09 (0.785), and 0.04 (0.001), respectively. There was a significant temporal difference (*P* <0.01) in stable fly trap counts by month with the highest trap counts in June (42.65 flies/trap) followed by May (25.11 flies/trap), and with much lower trap counts in July (8.91 flies/trap). Dairies that milked their cows 3 times/day had significantly (*P* = 0.01) higher trap counts (15.13 flies/trap) relative to dairies that milked 2 times/day (8.91 flies/trap). Trap counts were significantly (*P* < 0.01) higher when average weekly ambient temperature was ≤30°C (13.48 flies/trap) in comparison to when average weekly ambient temperature was >30°C (8.91 flies/trap) and were also significantly higher (*P* < 0.01) when the average weekly RH was <50% (8.91 flies/trap) in comparison to when average weekly RH ≥ 50% (5.12 flies/trap).

**Table 2:** Linear mixed model to estimate the association between the outcome (weekly log_10_ stable fly trap count) and explanatory variables study month, environmental factors, management practices and trap location on the study dairies.

Variable	Levels	Coefficient	Standard error	P-value	95% CI	
					Lower	Upper
Intercept		0.95	0.144	< 0.01	0.67	1.23
Month	July	Referent				
	May	0.45	0.036	<0.01	0.39	0.50
	June	0.68	0.026	<0.01	0.63	0.73
Milking times/day	2 times	Referent				
	3 times	0.23	0.091	0.01	0.05	0.40
Average weekly ambient temperature (°C)	>30 ⁰C	Referent				
	≤30 ⁰C	0.18	0.029	<0.01	0.12	0.24
Average weekly relative humidity (%)	<50%	Referent				
	≥50%	-0.24	0.033	<0.01	-0.31	-0.18
Addition of water to the TMR	No	referent				
	Yes	-0.09	0.072	0.17	-0.24	0.04
Interaction Term: Addition of water to the TMR X Average weekly relative humidity ≥ 50%		0.14	0.051	<0.01	0.04	0.24
Feed by-products*	None	Referent				
	AL	0.32	0.112	<0.01	0.10	0.55
	AL&W	0.37	0.119	<0.01	0.13	0.60
	AL,W&F	0.49	0.178	<0.01	0.14	0.84
	AL,F&V	0.56	0.224	0.01	0.12	1.00
	AL,W,F&V	0.60	0.160	<0.01	0.28	0.91
Fly control programs ǂ	0	Referent				
	1	-0.26	0.083	<0.01	-0.42	-0.09
	2	-0.14	0.128	0.25	-0.39	0.10
	3	-0.03	0.096	0.71	-0.22	0.15
Trap location	Middle	Referent				
	Periphery	0.19	0.062	<0.01	0.07	0.32

* Feed by-products are almond hulls (AL), wet distillers grain (W), fruits (F), and vegetables (V).

ǂ Fly control programs are either no stable fly control (0), insecticides applied to dairy facility by pest control company (1), insecticides applied directly to cattle (2), or dairies used parasitoid wasps and/or add larvicide in the TMR (3).

Addition of water to the TMR was only significantly (*P* < 0.01) associated with trap counts when RH was ≥ 50% (5.75 flies/trap), compared to dairies without water added to their TMR at the same RH (5.12 flies/trap). Feed by-products were grouped into 6 profiles by type including none added (2 dairies), only almond hulls (7 dairies), almond hulls and wet distillers’ grains (6 dairies), almond hulls, wet distillers’ grain, and fruits (one dairy), almond hulls, fruits, and vegetables (one dairy), or almond hulls, wet distillers’ grain, fruits, and vegetables (3 dairies).

The addition of feed by-products including almond hulls, wet distillers’ grain, fruits, and vegetables to the TMR in any combination was associated with significantly higher stable fly trap counts (*P* < 0.01) compared to dairies that did not provide any feed by-products (8.91 flies/trap). Specifically, feeding all 4 by-products was associated with the highest trap counts (35.48 flies/trap), followed by feeding almond hulls, fruits, and vegetable by-products (32.35 flies/trap), almond hulls, wet distillers grain and fruits (27.54 flies/trap), and then almond hulls and wet distillers grain (20.89 flies/trap). Amongst dairies that fed by-products, the lowest trap counts were for those that fed almond hulls only (18.62 flies/trap).

The fly control program variable at each dairy was categorized as 1) no stable fly control program (5 dairies), 2) company hired to apply insecticides to the entire facility (6 dairies), 3) company hired to apply insecticides only to the back and legs of cows exiting the milking parlor (6 dairies), and 4) use of parasitoid wasps and/or addition of a feed-through larvicide to TMR (3 dairies). Although some dairies utilized granular house fly baits to control house flies, the use of these baits was not considered a method for stable fly control as the baits are only effective to control house flies. Fly control categories 2 and 3 include methods to control adult stable flies while fly control category 4 includes methods to control immature stable fly development.

The model predicted a significant reduction (*P*<0.01) in the weekly stable fly trap counts on dairies that hire a company to spray the whole facility during the fly season (4.89 flies/trap) in comparison to dairies that had no stable fly control program (8.91flies/trap) accounting for other variables in the model. Other fly control programs (leg and back spray, using parasitoid wasps and/or adding larvicide to the TMR) were predicted to reduce the stable fly trap count (6.45 flies/trap and 8.31 flies/trap respectively), but the reduction was not significant (*P* > 0.05). Traps located on the periphery of the dairy had higher weekly stable fly counts (13.80 flies/trap) than traps in the middle of the dairy (8.91 flies/trap) after accounting for other variables in the model (*P* < 0.01). The remaining explanatory variables were not significantly associated with stable fly trap counts and hence dropped from the model.

## Discussion

Stable flies cause stress and discomfort to cattle affecting their behavior, welfare, and productivity, and stable fly biting activity is associated with bunching behavior in dairy cows [16]. Amongst the study months, leg and trap counts increased through May, peaked in June and then decreased through July. Specifically, mean leg counts peaked in the first week of June while trap counts peaked in the second week of June, and both leg and trap counts were lowest in the last week of July. Environmental factors including ambient temperature and RH had a significant effect on both leg counts and trap counts. Trap counts were increased by the addition of feed by-products to the TMR including almond hulls, wet distillers’ grain, fruits, and vegetables, while trap counts were decreased by the application of insecticidal sprays targeting adult stable flies applied across the entire dairy.

### Pen level leg count model

Leg counts were modeled to estimate the variability between pens based on the average leg count of 15 cows per pen. Our approach differs from estimating stable fly counts at the cow level as the latter would require cow-level variables such as location of cow in the pen, cow coat color, cow behavior during the count and may require observing the same cows over time. However, modeling stable fly counts at the cow level was not the objective of the current study. Instead, modeling pen level average leg counts can identify to actionable factors that producers can manage. Previous studies modeled leg counts as an average based on several cows or over time [5, 23, 24, 26, 39–42] as well as modeled fly counts for both leg counts and trap counts after log transformation [24, 26, 43].

### Temporality of stable fly activity

Stable flies are diurnal insects with the majority of their activity occurring between morning and afternoon [44–46]. Hence, for our study we modeled leg counts and trap counts using ambient temperature and relative humidity recorded between 9:00 AM to 11:00 AM, and 12:00 PM to 2:00 PM. Previous studies relied on use of data from the nearest weather stations to model the association between the stable fly count and ambient temperature and relative humidity [25, 28, 39, 47]. The current study used ambient temperature and relative humidity data obtained during the AM and PM counts from a mobile application (AccuWeather®). Mobile applications are the more likely source of weather data for producers compared to accessing a national database of nationwide weather stations to obtain local temperature and relative humidity. Both leg and trap counts showed a similar pattern of stable fly activity relative to study month (June>May>July). Our findings support previous studies that reported stable fly activity on California dairies was the highest in late May and early June, with activity decreasing in July as daytime temperature begins to peak [22, 23]. This temporal pattern in stable fly activity is reportedly driven by environmental factors, especially ambient temperature, and RH [24] with high abundance of stable flies often in response to spring rains [22]. Stable fly activity in California peaks earlier than it does in Nebraska where peak stable fly activity occurs in late June and early July, though as in California stable fly abundance is primarily a function of recent temperature and past precipitation [28]. In accordance with the stable fly activity pattern in California, the months of May and June are associated with higher odds of stable fly-avoidance behavior (bunching) compared to the month of July [16].

Leg and trap counts were negatively associated with ambient temperature and relative humidity (activity increased when temp ≤30⁰C or when RH <50%). A previous study reported that the maximum leg counts occurred in California at temperatures of 24⁰C and 30⁰C during spring and early summer [22]. Stable fly survival is reduced at temperatures above 30–35°C, with substantial reductions in adult longevity and fecundity [48–50] as well as greatly reduced survival of immature flies [3, 48, 49] and pupae [3] particularly as temperatures reach 35°C. Additionally, previous field studies in California show stable fly activity to be the highest during months when daytime temperatures generally remain below 30°C [16, 22]. Both biting activity and trap capture of stable flies is reduced during hours of the day when temperature exceeds 30–31°C [24, 43] when stable flies are reported to seek shaded resting locations [24]. Recently, it was observed that RH ≥ 50% was associated with lower odds of cow bunching [16].

### Effect of feed ingredients provided to cows on the stable fly (leg and trap counts)

Dairies that added agricultural by-products (almond hulls, wet distillers’ grain, fruits, and vegetables) to the TMR had higher weekly stable fly trap counts, perhaps as a result of providing additional decomposing organic matter that can provide suitable habitat for stable flies to lay their eggs [17, 21]. Additionally, almond hulls, fruits and some vegetables are rich in sugars which may serve as a carbohydrate food source for adult stable flies [51–57], hence possibly increasing adult stable fly survival and longevity. By-products are an attractive site for stable fly oviposition [20, 31]. Our results showed that stable fly count on traps, and not leg count, were significantly higher on dairies feeding by-products. The lack of significance of the variable by-products in the leg count model could be attributed to the smaller sample size of 10 dairies compared to 20 dairies in the trap count model. Alternatively, the amount of by-products stored at a single site (commodity barn) compared to that mixed in the TMR distributed in the feed bunker proximal to cows may explain why by-products had significant effect on the trap count and not leg count.

Addition of water to the TMR increased stable fly counts only when RH ≥ 50%, an effect modification (interaction) finding common to both leg and trap count models. While the current study design cannot determine the cause of such an interaction, others found that the addition of water to TMR composed primarily of haylage and silage resulted in a ration that was prone to spoilage at high temperatures [58]. Wet, decaying feed and other organic matter are good sites for stable flies to lay their eggs with silage and soiled straw bedding being common sites for stable fly development [19]. Furthermore, addition of water to the TMR may expedite secondary fermentation leading to the release of aromatic compounds that may be attractive to stable flies, while high RH might increase decomposition and molding of the feed, especially given that feed refusals are a good habitat for stable flies. Either or both of these mechanisms may attract biting flies which would mitigate the otherwise protective effect of having RH ≥ 50%. Proper fly management should focus on reducing the breeding sites for stable flies during spring and summer months to reduce stable fly abundance [59].

Lactating cow pens with cattle provided TMR containing straw had lower average leg count compared to pens provided TMR without straw. The high dry matter content of straw may reduce the overall water content in the TMR making it less suitable for immature stable fly development; in addition, straw has no sugars to provide a carbohydrate food source for adult stable flies. Similarly, inclusion of wheat silage in the TMR of dairy cows was associated with reduced leg count but only when ambient temperature was < 30 ⁰C. Wheat silage has a lower starch content than corn silage [61] so replacing corn silage with wheat silage in the TMR may result in a lower carbohydrate food source for adult stable flies thereby retaining fewer flies in the vicinity of the cattle pen [51, 52, 54–56]. Future research accounting for stable fly activity should explore impact of feed ingredients on stable fly abundance, preferably utilizing empirical study designs compared to the current observational study. However, data from the current study point at the potential for feed management during spring and summer as a tool to control stable flies on California dairies.

### Fly control programs

Only dairies that hired a pest control company to treat the entire dairy with an insecticide spray had reduced stable fly activity as determined by weekly trap counts. All other fly control methods, including application of fly sprays directly to cows exiting the milking parlor and use of parasitoid wasps and/or application of insect growth regulators in cow feed resulted in no significant reduction in stable fly activity. While our survey of dairy operators did not record the specific insecticidal products used by pest management companies hired to apply insecticides to the entire dairy, nearly all insecticides registered for stable fly control on cattle facilities are synthetic pyrethroids with a single organophosphorus insecticide (DIBROM 8) also still available for application to a dairy facility [60]. These insecticides are long-lasting, residual formulations that are applied to dairy facility structures and adjacent vegetation on which stable flies rest. Although some level of resistance to synthetic pyrethroids has recently been shown in California [61], its informative that the application of insecticides still reduced stable fly activity observed at the dairy level in our study herds.

### Limitations

Although the current study included a large number of dairies, the identified associations between stable fly activity with study month, ambient temperature, RH, and TMR components are based on an observational study. Hence, further studies are required to prove the causal association between addition of by-products (almond hulls, wet distillers’ grain, fruits, and vegetables), wheat silage, or water to dairy cattle TMR and stable fly activity. In addition, our findings indicate that fly control programs applied on the study dairies using insecticide sprays on facilities were associated with reduced stable fly counts on traps; however, further experimental studies are required to evaluate the efficacy of available fly control methods on the abundance of stable flies on cows as measured by leg counts.

The current study dairies are located in Tulare and Kings Counties, where the environmental factors such as the ambient temperature and RH may be different than other regions, hence, the generalizability of our results may be limited to areas with the same environmental conditions. Dairies included in the study were freestall and open lots which are the common dairy designs, but other facility designs such as pasture-based dairies may require further investigation.

## Conclusion

Our results showed that ambient temperature ≤30° C and RH <50%, were associated with increased stable fly activity as measured by both leg and Alsynite trap counts. The last two weeks of May and the first three weeks of June were associated with higher stable fly activity as measured by both leg and Alsynite trap counts in comparison to July. Feeding by-products; almond hulls, wet distillers’ grain, vegetables, and fruits was associated with higher numbers of stable flies on traps. In contrast, adding straw to the TMR was associated with lower leg counts. In addition, hiring a company to apply insecticides to the entire dairy facility for fly control resulted in lower trap counts.

Lactating cow pens bordered by trees had higher leg counts. This result is similar to that of [62] who found that traps positioned between cattle and a nearby tree line captured more stable flies than traps positioned near cattle without nearby vegetation. Presumably, these nearby trees provide a suitable resting site for stable flies allowing the flies to remain in the near vicinity of their cattle hosts. Traps on the periphery of the dairy captured more stable flies than those in the center of the dairy perhaps due to the increased presence of nearby resting locations for stable flies (trees, vegetation) which were generally more available at the dairy periphery. Also, stable flies in the center of the dairy are closer to cattle and may be relatively less attracted to the traps than flies at the dairy periphery further from cattle. In this study, leg counts exceeded two flies per leg for 4 weeks from May to July 2017 not quite reaching the economic threshold of 5 stable flies per leg where negative production impacts are anticipated [9]. However, since increased cattle bunching behavior is reported to occur even with >1 stable fly per leg [16], it is possible that production and economic impacts could be noted at even the leg counts reported in this study.

## Supporting information

S1 Data (XLSX)
